# The characterization and comorbidities of heterozygous Bardet-Biedl syndrome carriers

**DOI:** 10.7150/ijms.92766

**Published:** 2024-02-25

**Authors:** Meng-Hua Li, I-Chieh Chen, Hui-Wen Yang, Hsin-Chien Yen, Yung-Chieh Huang, Chia-Chi Hsu, Yi-Ming Chen, Yu-Yuan Ke

**Affiliations:** 1 Division of Pediatric Genetics and Metabolism, Children's Medical Center, Taichung Veterans General Hospital, Taichung, Taiwan.; 2 Department of Medical Research, Taichung Veterans General Hospital, Taichung, Taiwan.; 3 Department of Post-Baccalaureate Medicine, College of Medicine, National Chung Hsing University, Taichung, Taiwan.; 4 Division of Nephrology, Department of Pediatrics, Taichung Veterans General Hospital, Taichung, Taiwan.; 5 School of Medicine, National Yang Ming Chiao Tung University, Taipei, Taiwan.; 6 Division of Allergy, Immunology, and Rheumatology, Department of Internal Medicine, Taichung Veterans General Hospital, Taichung, Taiwan.; 7 Institute of Biomedical Science and Rong Hsing Research Center for Translational Medicine, National Chung Hsing University, Taiwan.

**Keywords:** Bardet-Biedl syndrome, BBS2 rs773862084, MKS1 rs199910690, heterozygotes, obesity, BMI, CKD

## Abstract

**Introduction:** Bardet-Biedl syndrome (BBS) is a rare autosomal recessive disorder with clinical features of retinal dystrophy, obesity, postaxial polydactyly, renal anomalies, learning disabilities, hypogonadism, and genitourinary abnormalities. Nevertheless, previous studies on the phenotypic traits of BBS heterozygous carriers have generated inconclusive results. The aim of our study was to investigate the impact of BBS heterozygosity on carriers when compared to non-carriers within the Taiwanese population.

**Materials and Methods:** This study follows a hospital-based case-control design. We employed the Taiwan Biobank version 2 (TWBv2) array to identify three specific loci associated with BBS (rs773862084, rs567573386, and rs199910690). In total, 716 patients were included in the case group, and they were compared to a control group of 2,864 patients who lacked BBS alleles. The control group was selected through gender and age matching at a ratio of 1:4. The association between BBS-related loci and comorbidity was assessed using logistic regression models.

**Results:** We found that BBS heterozygous carriers exhibited a significant association with elevated BMI levels, especially the variant rs199910690 in MKS1 (p=0.0037). The prevalence of comorbidities in the carriers' group was not higher than that in the non-carriers' group. Besides, the average values of the biochemistry data showed no significant differences, except for creatinine level. Furthermore, we conducted a BMI-based analysis to identify specific risk factors for chronic kidney disease (CKD). Our findings revealed that individuals carrying the CA/AA genotype of the BBS2 rs773862084 variant or the CT/TT genotype of the MKS1 rs199910690 variant showed a reduced risk of developing CKD, irrespective of their BMI levels. When stratified by BMI level, obese males with the MKS1 rs199910690 variant and obese females with the BBS2 rs773862084 variant exhibited a negative association with CKD development.

**Conclusion:** We found that aside from the association with overweight and obesity, heterozygous BBS mutations did not appear to increase the predisposition of individuals to comorbidities and metabolic diseases. To gain a more comprehensive understanding of the genetic susceptibility associated with Bardet-Biedl Syndrome (BBS), further research is warranted

## Introduction

Bardet-Biedl syndrome (BBS) is a rare autosomal recessive disorder characterized by gene pleiotropy. Its features include retinal dystrophy, obesity, postaxial polydactyly, renal anomalies, learning disabilities, hypogonadism, and genitourinary abnormalities^1^. In the general population, the prevalence of BBS is 0.7 per 100,000^2^, while the incidence varies among different regions, with a rate of 1 in 62,000 in Puerto Rico^3^, 1 in 18,000 in Newfoundland due to high consanguinity^4^, and 1 in 36,000 in Kuwait^5^. The occurrence of BBS in Europe is less frequent, affecting approximately 1 in 125,000 in the UK^6^. Among Asians, the incidence is notably lower, with rates as rare as 1 in 18 million^7^. As of July 31, 2023, there were a total of 45 confirmed BBS cases in Taiwan, as reported by the Health Promotion Administration, Ministry of Health and Welfare.

As BBS is a multisystem disorder, afflicted patients may commonly experience systemic effects, such as hypertension and metabolic abnormalities. To date, at least 26 genes associated with BBS have been identified^1^, ^8^, ^9^. These genes encode various components, including the BBSome complex, BBS-chaperonin complex, and other BBS proteins that function independently^10^. A number of studies have investigated the correlation between phenotype and genotype in BBS patients^9^. Forsythe *et al.* demonstrated the correlation between severe renal disease and BBS2, BBS10, and BBS12 patients^11^. In a study by Mujahid *et al.*, patients with BBS10 were at increased risk of metabolic syndrome^12^. However, previous studies regarding the characteristics of BBS heterozygous carriers have yielded inconclusive results. Clinically, the relatives of BBS patients are not predisposed to developing the diagnostic features. Croft *et al.* established an association between obesity and male BBS heterozygous carriers^13^. Benzinou *et al.* also presented evidence of increased obesity risk associated with BBS2, BBS4, and BBS6 genes^14^. Nevertheless, in a recent study, no correlation between obesity and metabolic disorders was found in first-degree relatives of BBS patients^15^. Based on prior studies, the attributes of BBS heterozygous carriers remain unclear.

This study aimed to evaluate the impact of BBS heterozygotes on carriers as compared to non-carriers within the Taiwanese population, focusing on the metabolic characteristics between genotype and phenotype. A second objective was to examine the variant's effect on different genders and its association with obesity. In this investigation, we aimed to uncover trends that could inform better genetic counseling and disease prevention for both affected BBS families and inadvertent carriers.

## Material and Methods

### Study design and data source

This retrospective case-control study made use of data from the Taiwan Precision Medicine Initiative (TPMI), a nationwide genetic program managed by Academia Sinica and partner hospitals. The TPMI cohort comprised Taiwanese participants from 16 hospitals across the country, with a significant portion of the cohort consisting of patients from Taichung Veterans General Hospital (TCVGH), a tertiary medical center. The study enrolled a total of 58,091 patients aged 18 years and older, who had visited 28 surgical and medical outpatient clinics at TCVGH between June 2019 and May 2021.

For our specific study cohort, we included a total of 3,580 patients whose genetic profiles were linked with medical claims data from TCVGH. This comprehensive dataset contained a wide range of information, including demographic characteristics, laboratory examinations, and diagnoses. The analysis was performed based on age and gender distribution. Diagnoses were coded using the International Classification of Diseases, Ninth and Tenth Revision, Clinical Modification (ICD-9-CM, ICD-10-CM) format. All of the participants provided informed consent and the study received approval from the Institutional Review Board (IRB No. SF19153A) of Taichung Veterans General Hospital's ethics committee. Written informed consent was obtained from all participants in accordance with the principles defined in the Declaration of Helsinki.

### Genotyping

In our study, blood samples were obtained from a total of 58,091 TPMI participants. Genomic DNA was isolated from these samples using DNA isolation kits (TIANGEN Biotech, Beijing, China). The extracted DNA was then quantified using a NanoDrop 2000 Spectrophotometer (Nano-Drop Technologies, Wilmington, DE, USA). We utilized the Taiwan Biobank version 2 (TWBv2) array to conduct next-generation sequencing (NGS) for the participants. This sequencing approach specifically targeted 114,000 risk variants in 2,831 unusual disease genes, which were carefully selected from reputable sources such as ClinVar, ACMG, GWAS Catalog, HGMD, locus-specific databases, and published literature^16^.

For rare variants genotyping and quality control, advanced normalization was performed to correct misclustering caused by the batch effect. This process was carried out using the advnorm package provided by Thermo Fisher Scientific (Santa Clara, CA, United States). Additionally, we applied a rare heterozygote adjustment to exclude probesets with inconsistent signals in replicated probes. The rare heterozygote adjustment was conducted using the axiomBestPractices-1.2.4 program, employing the "do-rare-het-adjustment” command^17^.

### Participants

After conducting a review of the literature, we selected three available loci associated with BBS matched with the Affymetrix Taiwan Biobank version 2 (TWBv2) array. There were two susceptibility alleles in BBS2 and one allele in MKS1, including rs773862084 (BBS2), rs567573386 (BBS2), and rs199910690 (MKS1). The MKS1 gene is not limited to Bardet-Biedl syndrome; it is also implicated in allelic disorders, such as Joubert syndrome and Meckel syndrome, which result in ciliary dysfunction^9^. A total of 716 patients who had variants associated with BBS were enrolled as the case group. A total of 2,864 patients without BBS alleles were matched to the case group by gender and age at a ratio of 1:4 to serve as the control group.

### Covariates

We obtained comorbidity information from the electronic health records of TCVGH based on ICD-9 and ICD-10 diagnostic codes. Comorbidities were identified, including hyperlipidemia (ICD-9-CM code 272, ICD-10-CM code E78.1-E78.5), hypertension (ICD-9-CM code 401-405, ICD-10-CM code I10-I15), obesity (ICD-9-CM code 278, ICD-10-CM code E66), diabetes mellitus (DM) (ICD-9-CM code 250, ICD-10-CM code E10.9 and E11.9), DM comorbidity with retinopathy, neuropathy, or chronic kidney disease (ICD-9 code 250, ICD-10-CM code E08 and E11), chronic kidney disease (CKD) (ICD-9-CM code 585 and ICD-10 code N18.1-N18.9), renal cancer (ICD-9-CM code 189, ICD-10-CM code C64 and C65), acute myocardial infarction(AMI) (ICD-9 code 410, ICD-10-CM code I21.3 and I24.9), coronary artery disease (CAD) (ICD-9 code 411-413, 414.00, 414.01 and ICD-10 code I20.8, I24.1, I25.1, I25.2), and cerebrovascular accident (CVA) (ICD-9 code 433-438 and ICD-10 code I63.5, I63.9, I67, I69). These comorbidities were identified if the diagnostic code was used once during admission or at least twice in the outpatient service.

Biochemical data included lipid profiles (low-density lipoprotein [LDL], high density lipoprotein [HDL], triglyceride [TG], and total cholesterol), blood sugar status (fasting glucose level and hemoglobin A1c [HbA1c]), serum insulin level, uric acid level, serum creatinine, and liver enzymes (alanine aminotransferase [ALT], aspartate aminotransferase [AST]). We selected the initial biochemical data collected from each individual in our hospital. Logistic regression was employed to examine the associations between BBS variants and the collected biochemical data.

### Statistics

Statistical analyses were carried out using the Statistical Package for the Social Sciences (SPSS) version 24.0 (Armonk, NY: IBM Corp.). Statistical significance was defined as p-values less than 0.05. The demographic information is presented as mean ± standard deviation (SD) for continuous variables and as number (percent) for categorical variables. To compare variables between BBS carriers and non-BBS carriers, Student's t-test was conducted for continuous variables, while Chi-square test was performed for categorical variables. Fisher's exact test was utilized to compare variables between alleles in BBS2 and MKS1. Multivariate logistic regression analysis was employed to calculate odds ratios (OR) and 95% confidence intervals (95% CI) for the three variants, adjusting for potential confounders. Furthermore, the impact of these variants on comorbidity was explored.

## Results

### Baseline characteristics and genetic variations associated with Bardet-Biedl Syndrome

**Figure 1:**
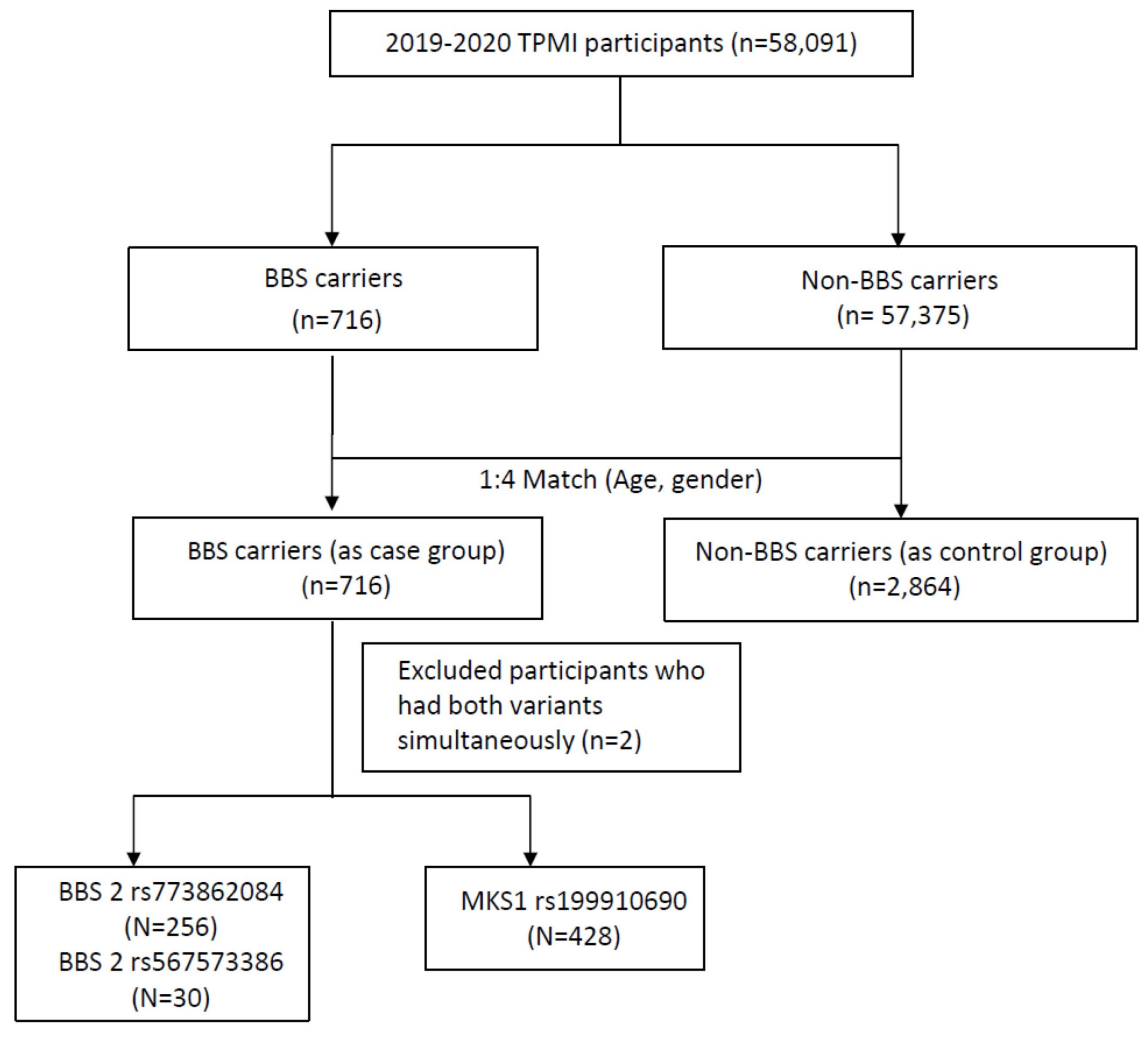
Study participants enrollment flow chart. BBS, Bardet-Biedl syndrome.

A total of 58,091 participants were included in the study, and the participant enrollment process is depicted in Figure 1. All recruited participants underwent genotyping. Among them, 716 individuals (1.23%) were identified as having susceptibility alleles associated with Bardet-Biedl syndrome (BBS carriers), while 57,375 individuals (98.77%) served as non-BBS carriers. After matching for age and gender at a 1:4 ratio, the BBS case group consisted of 716 subjects, and the control group consisted of 2,864 subjects. The baseline demographics of the study participants are shown in Table 1. Individuals with BBS variants exhibited higher percentages of overweight (BMI ≥ 24 kg/m^2^) compared to those without BBS variants (55.47% vs. 51.61%). The mean value of BMI in the BBS case group was 25.02±4.64 kg/m^2^ with significant differences observed. Conversely, hyperlipidemia (32.4% vs. 39.49%, p = 0.0005), hypertension (26.96% vs. 37.26%, p<0.0001), diabetes mellitus (DM) (25.28% vs. 34.67%, p<0.0001), diabetes mellitus comorbidity (4.47% vs. 7.93%, p = 0.0014), and chronic kidney disease (CKD) (18.72% vs. 36.94%, p<0.0001) were inversely associated with individuals without BBS variants, as they were more prevalent in the control group.

Furthermore, in Table 2, individuals with BBS variants displayed higher levels of the liver enzyme alanine aminotransferase (ALT) (32.42 ± 38.16 vs. 27.49 ±27.94 uIU/mL, p = 0.0026) and demonstrated better renal function, as indicated by the lower levels of serum creatinine (1.17 ± 1.64 vs. 1.66 ± 2.32 uIU/mL, p<0.0001) compared to their counterparts. Additionally, participants carrying BBS susceptibility alleles exhibited higher insulin levels (46.97 ± 119.36 vs. 17.69 ± 23.14 uIU/mL) and elevated levels of aspartate aminotransferase (AST) (30.94 ± 44.72 vs. 26.66 ± 34.87 uIU/mL), although the latter difference was not statistically significant.

### Comparisons of comorbidities and serology among each of the BBS risk alleles

Within the BBS carriers group, among the 716 participants with BBS variants, two cases were identified as double heterozygous carriers, possessing both BBS2 rs773862084 and MKS1 rs199910690 heterozygous mutations. The remaining 714 subjects were heterozygous carriers, each having a single copy of the mutated gene. Specifically, among the BBS2 variants, 286 patients (40.06%) had rs773862084 (256 subjects) or rs567573386 (30 cases), while the remaining 428 patients (59.94%) had MKS1 variants with rs199910690. The characteristics of comorbidities and serology in each of the BBS risk alleles are shown in Table 3 and Table 4.

For the majority of risk alleles in the BBS group, no statistically significant associations were found with any of the comorbidities. However, individuals carrying the MKS1 rs199910690 allele exhibited a noteworthy increase in the incidence of cerebrovascular accidents (14.49%, p = 0.0009). Furthermore, although the associations did not reach statistical significance, patients with the MKS1 rs199910690 allele showed the highest proportions of obesity (BMI > 27 kg/m^2^), hyperlipidemia, hypertension, diabetes mellitus, diabetes mellitus comorbidity, CKD, renal cancer, and CAD. Therefore, in Table 5, we subsequently conducted a comparison of the BMI levels between the single variant and the control group, respectively. The variant rs199910690 in MKS1 revealed an increased risk of overweight and obesity. Regarding the mean values of the biochemistry data, no significant differences were observed.

### BMI associated with genetic variants

In order to examine the specific risk factors for each comorbidity based on BMI, we performed univariate analyses for hyperlipidemia, DM, hypertension (refer to Supplementary Tables 1-3), and CKD (Table 6). As shown in Supplementary Tables 1-3, it can be seen that males have a higher risk of developing diseases when stratified by BMI, as compared to females. The study revealed a significant reduction in the risk of hyperlipidemia, DM and hypertension among individuals carrying the rs773862084 alleles when BMI was <24 kg/m2. When stratified by BMI, all three genetic variants demonstrated protective associations with each comorbidity, except for coronary artery disease (CAD). In Table 6, the BBS2 rs773862084 CA/AA genotype demonstrated a lower risk of CKD compared to the CC genotype, with an OR of 0.45 (95% CI: 0.325-0.615, p < 0.0001). Similarly, the MKS1 rs199910690 CT/TT genotype was also associated with a reduced risk of CKD when compared to the CC genotype, with an OR of 0.43 (95% CI: 0.331-0.549, p < 0.0001).

### BMI and genetic variants in different genders

We further divided participants according to different gender and BMI levels, as presented in Table 7a and Table 7b. We conducted a multivariate logistic regression analysis, to explore the relationship between genetic variants, BMI levels, and CKD. Additionally, potential confounding factors, such as diabetes mellitus (DM) and hyperlipidemia, were adjusted for in the analysis. Our findings found a statistically significant association indicating protection against the development of CKD in both males and females who carry either the BBS2 rs773862084 or MKS1 rs199910690 variant, when compared to their respective counterparts.

Specifically, among participants with BMI > 27 kg/m^2^, obese males carrying the MKS1 rs199910690 variant exhibited a negative association with the development of CKD (OR: 0.25, 95% CI: 0.123-0.523, p = 0.0002). Similarly, obese females carrying the BBS2 rs773862084 variant had a lower risk of developing CKD (OR: 0.09, 95% CI: 0.012-0.073, p = 0.0239).

Furthermore, in Supplementary Tables 4 and 5, these obese females carrying the BBS2 rs773862084 variant also showed a significant inverse association with development of hyperlipidemia (OR: 0.34, 95% CI: 0.113 - 0.994, p = 0.0487). For men with various BMI levels, the difference in developing hyperlipidemia was not statistically significant. As for developing diabetes mellitus (DM), presented in Supplementary Tables 7 and 8, we observed a statistically significant protective association in males who carried the MKS1 rs199910690 variant, indicating a protective effect. However, when considering different BMI levels and genders, we did not find any statistically significant associations with the development of diabetes mellitus (DM).

## Discussion

Our results demonstrate that BBS heterozygous carriers had increased BMI levels, particularly in individuals with the variant rs199910690 in MKS1. In this study, we investigated the distinct genetic risk variants for CKD, stratified by BMI levels. Our findings revealed that individuals carrying the CA/AA genotype of the BBS2 rs773862084 variant or the CT/TT genotype of the MKS1 rs199910690 variant did not exhibit an increased risk of developing CKD, irrespective of their BMI level. Moreover, among participants classified as obese (BMI > 27 kg/m²), males with the MKS1 rs199910690 variant and females with the BBS2 rs773862084 variant displayed a negative association with the development of CKD. Our dataset focused on the phenotype-genotype relationship of BBS heterozygous carriers in a hospital-based case-control study conducted in Taiwan. Notably, this study is the first to compare these associations within an Asian population. We sincerely hope that these results can be employed in the future to provide enhanced health guidance for individuals with the known variants, thereby offering valuable insights from a disease prevention perspective.

As is widely known, obesity is a prevalent manifestation in BBS patients, with approximately 89% of diagnosed individuals noted to have obesity^9^. For example, in individuals with BBS1, the prevalence of obesity is significantly higher compared to both carriers and non-carriers of the BBS1 gene^18^. Despite mostly having normal birth body weight, BBS patients experience rapid body weight gain during childhood. Among children and adolescents aged 2 to 19 years who were enrolled in the Clinical Registry Investigating Bardet-Biedl Syndrome (CRIBBS), the prevalence of overweight (BMI > 25 kg/m^2^) or obesity (BMI > 30 kg/m^2^) in BBS patients ranges from 86% to 95%^19^. With respect to adults, in a study by Mujahid *et al.*, the average BMI of 152 BBS patients aged 16 to 58 years in United Kingdom was 35.7 ± 7.8 kg/m^2^^12^. The Asian-specific BMI cutoffs for overweight and obesity was relatively low in comparison to White adults. Compared to individuals of the same age, sex, and BMI among white populations, Asians typically exhibit a higher percentage of body fat. Asians are known to have elevated risks of developing type 2 diabetes and cardiovascular disease, even when their BMI is below the WHO-defined cutoff point of 25 kg/m² for overweight 20, 21. As suggested by W. H. Pan *et al.*^22^, for Taiwanese individuals, a BMI of 23.4 could be the threshold for optimizing both positive and negative predictive values for comorbidities. The BBS proteins, namely, ciliary‑related proteins, play a crucial role in ciliary function by directing vesicle trafficking to the ciliary membrane. When cilia dysfunction occurs, it can lead to hyperleptinemia and leptin resistance. The disruption in leptin action further hampers the proopiomelanocortin (POMC) neurons in the arcuate nucleus of the hypothalamus. This is followed by the inactivation of the melanocortin-4 receptor (MC4R), which results in hyperphagia (increased appetite) and ultimately contributes to obesity^23^. Patients with BBS were known to have hyperleptinemia with leptin resistance compared with individuals without BBS^24^. Benzinou *et al.* reported that variants in BBS2, BBS4, and BBS6 showed evidence of association with common obesity in French Caucasians^14^. In a study by Day *et al.*, rs59252892 in BBS9 exhibited a statistically significant association with obesity. It has been hypothesized that these BBS9 variants may lead to a subtle increase in the shuttling of LEPR (leptin receptor), resulting in leptin resistance^25^. Therefore, obesity is recognized as a hallmark of BBS. Individuals with BBS also encounter the challenges associated with metabolic syndrome, that is, obesity related complications including hyperlipidemia, insulin resistance and fasting hyperglycemia^9^. In a study by Mujahid *et al.*, the prevalence of metabolic syndrome among BBS patients was 54.3%^12^. For individuals with BBS, an annual assessment of fasting blood glucose, HbA1c, and lipid levels (triglycerides, HDL-C, LDL-C, and total cholesterol) is recommended. Additionally, for better quality of life, reduced-calorie diet and lifestyle changes such as regular exercise program are suggested^8^. For pharmaceutical management, it is noteworthy that setmelanotide, a melanocortin-4 receptor (MC4R) agonist, was the first drug approved by the United States Food and Drug Administration (FDA). It functions by binding to the melanocortin-4 receptor (MC4R), thereby reducing appetite and increasing energy expenditure. It is specifically indicated for the treatment of obesity resulting from genetic disorders such as proopiomelanocortin (POMC), proprotein convertase subtilisin/kexin type 1 (PCSK1), and leptin receptor (LEPR) deficiency^26^, ^27^. In the clinical trial by Forsythe *et al.*^28^, individuals with BBS and obesity experienced a significant reduction in BMI levels and an improvement in health-related quality of life after 52 weeks of setmelanotide therapy. However, the efficacy of setmelanotide in patients with heterozygous variants remains inconclusive, as ongoing clinical trials focusing on heterozygous participants are underway^29^-^31^. Adverse effects associated with setmelanotide treatment included injection site reactions, skin hyperpigmentation, sexual dysfunction, and psychological issues such as depression and suicidal ideation^27^. In a study by Robert *et al.*^32^, all BBS participants who received setmelanotide treatment experienced injection site reactions, with eighty percent developing hyperpigmentation.

In our study, we verified that adult individuals who were BBS heterozygous carriers exhibited higher percentages of elevated BMI level compared to those without BBS variants, which is consistent with findings from previous studies^13^, ^14^. With regard to the risk allele in the BBS group, more specifically in our study, the variant rs199910690 in MKS1 showed evidence of associations with overweight and obesity. Conversely, our investigation did not reveal any significant links between individuals carrying rs773862084 variants in BBS2 and overweight or obesity. This outcome contrasts with the findings reported by Benzinou *et al.*, which suggested an association between BBS2 and obesity. We postulate that this difference might be attributed to the relatively limited number of participants in our study or the presence of diverse SNPs encoding proteins with varying functions. Furthermore, concerning BBS heterozygous carriers, prior reports have indicated that the incidence rates of hypertension and diabetes were similar when comparing carriers and non-carriers^15^. Our study aligns with these findings, as it revealed no significant disparities in the risk of metabolic diseases and comorbidities, which include hyperlipidemia, hypertension, diabetes mellitus, diabetes mellitus comorbidity, chronic kidney disease (CKD), acute myocardial infarction (AMI), coronary artery disease (CAD), and cerebrovascular accident (CVA). Furthermore, it is noteworthy that our results were not solely concerned with the risk of developing the disease, but our biochemical analysis also yielded consistent outcomes. This implies that the lipid levels and blood sugar homeostasis in BBS heterozygous carriers were comparable to those of the control groups.

Kidney disease is also a major features of BBS, encompassing structural anomalies, urologic complications, and chronic kidney disease (CKD). The prevalence of kidney disease in BBS is 52%, with chronic kidney disease (CKD) being a prominent contributor to both morbidity and mortality in individuals with BBS^9^. Several studies have explored the genotype‐phenotype analysis in Bardet‐Biedl syndrome. In a study by Niederlova *et al.*, a strong correlation between genotype and renal anomalies was observed among individuals with BBS. Specifically, patients carrying mutations in the core BBSome subunits, such as BBS2, BBS7, or BBS9, exhibited a relatively high frequency of renal anomalies^33^. Forsythe *et al.* demonstrated that mutations in BBS2, BBS10, and BBS12 were more likely to be associated with severe renal disease. Among these, patients with BBS10 mutations were more frequently observed as the stage of CKD increased. The study further provided valuable findings insights that CKD4-5 mainly develops during childhood; otherwise, individuals in adulthood have a comparatively lower risk of experiencing severe renal disease^11^. Being ciliopathies, the renal impairment in BBS primarily arises from tubular ciliary dysfunction. This dysfunction disrupts the mammalian target of rapamycin(mTOR) signaling pathway and eventually contributes to the development of cystic kidney disease^10^, ^11^, ^34^, ^35^. Of note, a significant association was found between renal ciliary dysfunction and CKD. Using electron microscopy, Imhoff *et al.* also observed thickening of the basement membrane in BBS patients, which subsequently led to hypertension or diabetes^36^. There was no consensus regarding the risk of renal disorders in heterozygous carriers when compared to the general population. Concerning renal cancer differing opinions exist. A study by Beales *et al.* reported 3 cases of renal cancer among 180 parents of BBS patients, implying a 17-fold increased risk compared to the general population^37^. However, Hjortshøj *et al.* surveyed 428 relatives of BBS patients and found no elevated risk of renal cancer^38^. Our results also indicated no increased risk of developing renal cancer. Individuals carrying a single mutation variant of Bardet-Biedl syndrome, known as obligate carriers, were not prone to having renal function impairment. As previously noted by MP Webb *et al.*, no significant difference in the incidence of stage-3 CKD was observed when comparing carriers and non-carriers, which was consistent with our results^15^. We demonstrated that the renal function of individuals with BBS variants was not inferior to that of the control group. Additionally, we established that the risk of CKD in heterozygous carriers with the BBS2 rs773862084 CA/AA genotype or the MKS1 rs199910690 CT/TT genotype was not higher compared to non-carriers, regardless of the BMI level. Obesity is a well-documented risk factor for chronic kidney diseases^39^. Overweight persons (25≤BMI<30) had an elevated risk for chronic kidney diseases, and persons with higher BMI (>30) had an even higher risk^40^. The findings were similar to those found in a Taiwanese population^41^. Renal hyperfiltration can occur in overweight or obese patients, resulting in an overestimation of renal function test with serum creatinine or cystatin C^42^. According to findings reported by Basolo *et al.*, obese individuals were observed to have glomerular hyperfiltration, subsequently leading to an elevated creatinine clearance^43^. In our study, where we identified a risk of developing overweight and obesity in the BBS heterozygous carriers group, we also noted that the creatinine levels in the carriers' group were lower than those in the non-carriers' group, with significant differences observed. Nevertheless, renal hyperfiltration can only partially explain our finding that BBS heterozygous carriers have lower serum creatinine level and a lower rate of CKD. Whether partial mutation in BBSome subunits can lead to renal protection requires further research using experimental cell or animal models.

There were some limitations in our study. First, certain detailed information was not recorded in the study. There were several genes identified for obesity which could be classified as monogenic obesity and polygenic obesity^44^. Lifestyle and dietary habits are known to contribute to obesity as well. We were unable to comprehensively evaluate the potential confounders. Regarding the renal function evaluation, we did not conduct a complete assessment of renal structural anomalies and the severity of proteinuria in our study. Second, it is possible that we underestimated the impact of genetic variants on the BBS heterozygous carriers because of the selection bias inherent in hospital-based research. Third, the enrolled participants were exclusively East Asians, and therefore our conclusions may not be applicable to genetic studies involving other ethnic groups. Lastly, due to the rarity of BBS, the allele frequency remained relatively low, ranging from 1/250 to 1/2200^45^, resulting in a low number of participants in our study. Longer-term prospective research is required to further confirm the consistent effect of the genetic variant on the general population. We recommend initiating a thorough management and monitoring approach for disease prevention as it could benefit BBS heterozygous carriers.

## Conclusion

Our study revealed that individuals carrying heterozygous mutations in the MKS1 gene, specifically the rs199910690 variant associated with Bardet-Biedl syndrome, had an elevated risk of overweight and obesity. However, regardless of their body mass index (BMI), individuals possessing the CA/AA genotype for BBS2 rs773862084 or the CT/TT genotype for MKS1 rs199910690 did not exhibit an increased risk of developing chronic kidney disease (CKD). Furthermore, within the subset of participants with a BMI of > 27 kg/m², males carrying the MKS1 rs199910690 variant and females carrying the BBS2 rs773862084 variant demonstrated a negative correlation with the development of CKD. These findings underscore the need for further research to comprehensively explore the genetic susceptibility factors associated with Bardet-Biedl syndrome.

## Supplementary Material

Supplementary tables.

## Figures and Tables

**Table 1:** Basic characteristics of the study population.

Variables	With BBS (N=716)						Without BBS (N=2,864)		*P* value
	N	%				N		%	
**Age, years (mean/SD)^a^**	59.02	15.75			59.02			15.74	1
**Gender (n, %)^b^**									1
Female	380	53.07%			1520			53.07%	
Male	336	46.93%			1344			46.93%	
**BMI (n, %)^b^**									
BMI (Mean/SD)	25.02	4.64			24.59			5.65	0.0419
BMI<24 kg/m^2^	293	44.53			1305			48.39	0.0054
24≦BMI≦27 kg/m^2^	171	25.99			760			28.18	
BMI>27 kg/m^2^	194	29.48			632			23.43	
**Comorbidities (n, %)^b^**									
Hyperlipidemia									0.0005
No	484	67.6			1733			60.51	
Yes	232	32.4			1131			39.49	
Hypertension									<0.0001
No	523	73.04			1797			62.74	
Yes	193	26.96			1067			37.26	
Obesity									0.2715
No	705	98.46			2834			98.95	
Yes	11	1.54			30			1.05	
DM									<0.0001
No	535	74.72			1871			65.33	
Yes	181	25.28			993			34.67	
DM comorbidity*									0.0014
No	684	95.53			2637			92.07	
Yes	32	4.47			227			7.93	
CKD									<0.0001
No	582	81.28			1806			63.06	
Yes	134	18.72			1058			36.94	
Renal cancer									0.1751
No	708	98.88			2811			98.15	
Yes	8	1.12			53			1.85	
AMI									0.5151
No	706	98.6			2814			98.25	
Yes	10	1.4			50			1.75	
CAD									0.3278
No	603	84.22			2368			82.68	
Yes	113	15.78			496			17.32	
CVA									0.2599
No	638	89.11			2508			87.57	
Yes	78	10.89			356			12.43	
									

^a^ Continuous variables were expressed as mean ± standard deviation (SD) and were analyzed using Student's t-test for normal data distributions. ^b^ Categorical variables were expressed as numbers (percent) and were analyzed using the Chi-square test. *DM comorbidity with retinopathy, neuropathy, or chronic kidney disease Abbreviations: BBS: Bardet-Biedl syndrome; BMI: Body mass index; DM: diabetes mellitus; CKD: chronic kidney disease; AMI: acute myocardial infarction; CAD: coronary artery disease; CVA: cerebrovascular accident

**Table 2:** Basic biochemistry characteristics of the study population.

Variables	With BBS (N=716)						Without BBS (N=2,864)		*P* value
	Mean	SD				Mean		SD	
Biochemistry (mean/SD)^a^									
LDL (mg/dL)	118.25	36.73			117.38			42.85	0.6682
HDL (mg/dL)	52.72	15.79			54			16.8	0.2557
Triglyceride (mg/dL)	137.8	101.15			145.24			149.8	0.1848
Total cholesterol (mg/dL)	191.44	43.7			189.5			48.35	0.4367
Uric acid (mg/dL)	6.38	2.02			6.5			2.18	0.3376
Fasting glucose (mg/dL)	117.1	42.79			117.93			47.13	0.7218
HbA1c (%)	6.58	1.73			6.67			2.04	0.3666
Insulin (uIU/mL)	46.97	119.36			17.69			23.14	0.3597
Creatinine (mg/dL)	1.17	1.64			1.66			2.32	<0.0001
ALT (U/L)	32.42	38.16			27.49			27.94	0.0026
AST (U/L)	30.94	44.72			26.66			34.87	0.053
									

^a^ Continuous variables were expressed as mean ± standard deviation (SD) and were analyzed using Student's t-test for normal data distributions. Abbreviations: BBS: Bardet-Biedl syndrome; SD: standard deviation; LDL: low density lipoprotein; HDL: high density lipoprotein; HbA1c: glycosylated hemoglobin; eGFR: estimated glomerular filtration rate; ALT: alanine aminotransferase; AST: aspartate transferase.

**Table 3:** Comparisons of comorbidities among each of the BBS risk alleles.

Variables	rs773862084 (N=256)			rs567573386 (N=30)			rs199910690 (N=428)			*P* value
	N	%		N	%		N	%		
**Age, years (mean/SD)^a^**	59.1	15.71		57.63	15.11%		59.07	15.85		0.8852
**Gender (n, %)^b^**										0.582
Female	131	51.17		18	60		231	53.97		
Male	125	48.83		12	40		197	46.03		
**BMI (n, %)^b^**										0.2507
BMI<24 kg/m^2^	111	47.23		18	60		163	41.69		
24≦BMI≦27 kg/m^2^	57	24.26		7	23.33		107	27.37		
BMI>27 kg/m^2^	67	28.51		5	16.67		121	30.95		
**Comorbidities (n, %)^b^**										
Hyperlipidemia										0.1283
No	180	70.31		24	80		279	65.19		
Yes	76	29.69		6	20		149	34.81		
Hypertension										0.0978
No	193	75.39		26	86.67		303	70.79		
Yes	63	24.61		4	13.33		125	29.21		
Obesity										0.3842
No	254	99.22		29	96.67		420	98.13		
Yes	2	0.78		1	3.33		8	1.87		
DM										0.4185
No	195	76.17		25	83.33		315	73.6		
Yes	61	23.83		5	16.67		113	26.4		
DM comorbidity*										0.8593
No	246	96.09		29	96.67		408	95.33		
Yes	10	3.91		1	3.33		20	4.67		
CKD										0.9609
No	208	81.25		25	83.33		348	81.31		
Yes	48	18.75		5	16.67		80	18.69		
Renal cancer										^c^0.7994
No	254	99.22		30	100		422	98.6		
Yes	2	0.78		0	0		6	1.4		
AMI										^c^0.3959
No	252	98.44		29	96.67		423	98.83		
Yes	4	1.56		1	3.33		5	1.17		
CAD										0.1517
No	224	87.5		26	86.67		351	82.01		
Yes	32	12.5		4	13.33		77	17.99		
CVA										0.0009
No	241	94.14		29	96.67		366	85.51		
Yes	15	5.86		1	3.33		62	14.49		

^a^ Continuous variables were expressed as mean ± standard deviation (SD) and used ANOVA test for continuous variables. ^b^ Categorical variables were expressed as numbers (percent) and were analyzed using Chi-square test for categorical variables. ^c^ Using Fisher's exact test. Excluded 2 participants with two variants *DM comorbidity with retinopathy, neuropathy, or chronic kidney disease Abbreviations: BBS: Bardet-Biedl syndrome; BMI: Body mass index; DM: diabetes mellitus; CKD: chronic kidney disease; AMI: acute myocardial infarction; CAD: coronary artery disease; CVA: cerebrovascular accident.

**Table 4:** Comparisons of serology among each of the BBS risk alleles.

Variables	rs773862084 (N=256)			rs567573386 (N=30)			rs199910690 (N=428)			*P* value^a^
	Mean	SD		Mean	SD		Mean	SD		
Biochemistry										
LDL (mg/dL)	118.07	36.1		124.93	54.78		117.89	36.09		0.7707
HDL (mg/dL)	52.11	15.05		54.89	14.09		52.89	16.42		0.853
Triglyceride (mg/dL)	139.64	112.9		128.47	121.41		137.32	92.9		0.8954
Total cholesterol (mg/dL)	190.24	42.51		192.88	76.56		192.06	41.77		0.9198
Uric acid (mg/dL)	6.4	1.99		6.25	2.04		6.37	2.04		0.9625
Fasting glucose (mg/dL)	117.34	40.03		101.21	27.94		117.29	43.48		0.2628
HbA1c (%)	6.54	1.82		6.22	1.55		6.61	1.67		0.6458
Insulin (uIU/mL)	29.69	38.84		-	-		51.29	133.25		0.7907
Creatinine (mg/dL)	1.33	2.15		0.92	0.31		1.11	1.37		0.443
ALT (U/L)	35.92	54.15		31.41	21.85		30.47	26.43		0.2485
AST (U/L)	33.22	55.44		36.58	29.64		29.17	37.81		0.5579

^a^ Using ANOVA test for continuous variables. Excluded 2 participants with two variants Abbreviations: BBS: Bardet-Biedl syndrome; LDL: low density lipoprotein; HDL: high density lipoprotein; HbA1c: glycosylated hemoglobin; eGFR: estimated glomerular filtration rate; ALT: alanine aminotransferase; AST: aspartate transferase.

**Table 5:** Comparisons of the BMI level between carrier and non-carrier group, with variant BBS2 rs773862084 and MKS1 rs199910690 respectively.

Variables	BBS2 rs773862084 (N=256)		Non-carrier (n=2864)		*P* value		MKS1 rs199910690 (N=428)		Non-carrier (n=2864)		*P* value
	N	%	N	%			N	%	N	%	
BMI<24 kg/m^2^	111	47.23	1305	48.39	0.166		163	41.69	1305	48.39	0.0037
24≦BMI≦27 kg/m^2^	57	24.26	760	28.18			107	27.37	760	28.18	
BMI>27 kg/m^2^	67	28.51	632	23.43			121	30.95	632	23.43	

Abbreviations: BMI: Body mass index

**Table 6:** Stratification by BMI and CKD.

Variables	CKD											
	BMI<24 kg/m^2^			24≦BMI≦27 kg/m^2^			BMI>27 kg/m^2^			Overall		
	OR	95% CI	*P* value	OR	95% CI	*P* value	OR	95% CI	*P* value	OR	95% CI	*P* value^a^
Age, years												
Gender												
Female	1.00	─	─	1.00	─	─	1.00	─	─	1.00	─	─
Male	1.92	1.549-2.386	<0.0001	1.56	1.183-2.063	0.0017	1.49	1.106-1.995	0.0086	1.69	1.469-1.944	<0.0001
rs773862084												
CC	1.00	─	─	1.00	─	─	1.00	─	─	1.00	─	─
CA / AA	0.52	0.352-0.823	0.0053	0.43	0.221-0.846	0.0144	0.35	0.184-0.662	0.0013	0.45	0.325-0.615	<0.0001
rs567573386												
GG	1.00	─	─	1.00	─	─	1.00	─	─	1.00	─	─
GA / AA	0.24	0.056-1.066	0.0608	0.76	0.146-3.913	0.7378	0.43	0.048-3.895	0.4554	0.40	0.152-1.042	0.0607
rs199910690												
CC	1.00	─	─	1.00	─	─	1.00	─	─	1.00	─	─
CT / TT	0.49	0.327-0.718	0.0003	0.35	0.204-0.584	<0.0001	0.34	0.207-0.547	<0.0001	0.43	0.331-0.549	<0.0001

^a^ Comparisons of categorical variables were analyzed using logistic regression adjusted by age and gender. Abbreviations: CI: confidence interval; OR: odds ratio; BMI: Body mass index; CKD: chronic kidney disease

**Table 7a:** Stratification by BMI and CKD in females.

Variables	Female											
	BMI<24 kg/m^2^			24≦BMI≦27 kg/m^2^			BMI>27 kg/m^2^			Overall		
	OR^b^	95% CI	*P* value	OR	95% CI	*P* value	OR	95% CI	*P* value	OR	95% CI	*P* value^a^
rs773862084												
CC	1.00	─	─	1.00	─	─	1.00	─	─	1.00	─	─
CA / AA	0.46	0.218-0.949	0.0358	0.37	0.119-1.163	0.089	0.09	0.012-0.730	0.0239	0.38	0.218-0.654	0.0005
Age, years	1.02	1.008-1.028	0.0003	1.02	0.999-1.035	0.0697	1.03	1.015-1.053	0.0004	1.02	1.012-1.028	<0.0001
DM												
No	1.00	─	─	1.00	─	─	1.00	─	─	1.00	─	─
Yes	1.19	0.829-1.709	0.3451	1.11	0.658-1.857	0.7041	1.16	0.664-2.041	0.5963	1.15	0.891-1.472	0.2907
hyperlipidemia												
No	1.00	─	─	1.00	─	─	1.00	─	─	1.00	─	─
Yes	2.43	1.769-2.333	<0.0001	3.91	2.377-6.419	<0.0001	2.15	1.221-3.789	0.0081	2.70	2.131-3.411	<0.0001
												
rs199910690												
CC	1.00	─	─	1.00	─	─	1.00	─	─	1.00	─	─
CT / TT	0.45	0.266-0.769	0.0034	0.22	0.073-0.639	0.0056	0.53	0.258-1.099	0.0884	0.45	0.308-0.657	<0.0001
Age, years	1.02	1.008-1.028	0.0004	1.02	0.998-1.035	0.0772	1.04	1.016-1.054	0.0002	1.02	1.013-1.028	<0.0001
DM												
No	1.00	─	─	1.00	─	─	1.00	─	─	1.00	─	─
Yes	1.18	0.824-1.700	0.362	1.11	0.657-1.882	0.6938	1.10	0.629-1.924	0.7367	1.14	0.887-1.469	0.3023
hyperlipidemia												
No	1.00	─	─	1.00	─	─	1.00	─	─	1.00	─	─
Yes	2.52	1.832-3.453	<0.0001	3.85	2.326-6.377	<0.0001	2.30	1.309-4.030	0.0037	2.79	2.204-3.532	<0.0001

^a^ Comparisons of categorical variables were analyzed using logistic regression. ^b^ OR was adjusted for all variables in the table. Abbreviations: CI: confidence interval; OR: odds ratio; BMI: Body mass index; CKD: chronic kidney disease; DM: diabetes mellitus.

**Table 7b:** Stratification by BMI and CKD in males.

Variables	Male											
	BMI<24 kg/m^2^			24≦BMI≦27 kg/m^2^			BMI>27 kg/m^2^			Overall		
	OR^b^	95% CI	*P* value	OR	95% CI	*P* value	OR	95% CI	*P* value	OR	95% CI	*P* value^a^
rs773862084												
CC	1.00	─	─	1.00	─	─	1.00	─	─	1.00	─	─
CA / AA	0.54	0.271-1.075	0.0794	0.43	0.176-1.04	0.0609	0.51	0.239-1.086	0.0809	0.50	0.324-0.772	0.0018
Age, years	1.02	1.008-1.032	0.0014	1.03	1.015-1.043	<0.0001	1.04	1.020-1.050	<0.0001	1.03	1.02-1.035	<0.0001
DM												
No	1.00	─	─	1.00	─	─	1.00	─	─	1.00	─	─
Yes	1.23	0.812-1.851	0.3326	1.45	0.987-2.141	0.058	1.86	1.231-2.806	0.0032	1.53	1.219-1.913	0.0002
hyperlipidemia												
No	1.00	─	─	1.00	─	─	1.00	─	─	1.00	─	─
Yes	3.21	2.167-4.745	<0.0001	1.83	1.248-2.681	0.002	1.84	1.219-2.779	0.0037	2.24	1.794-2.796	<0.0001
												
rs199910690												
CC	1.00	─	─	1.00	─	─	1.00	─	─	1.00	─	─
CT / TT	0.53	0.268-1.062	0.0737	0.40	0.211-0.762	0.0053	0.25	0.123-0.523	0.0002	0.38	0.262-0.550	<0.0001
Age, years	1.02	1.007-1.032	0.0016	1.03	1.015-1.043	<0.0001	1.04	1.020-1.051	<0.0001	1.03	1.020-1.035	<0.0001
DM												
No	1.00	─	─	1.00	─	─	1.00	─	─	1.00	─	─
Yes	1.24	0.818-1.872	0.312	1.46	0.990-2.154	0.0563	1.72	1.128-2.609	0.0117	1.50	1.196-1.884	0.0005
hyperlipidemia												
No	1.00	─	─	1.00	─	─	1.00	─	─	1.00	─	─
Yes	3.16	2.135-4.683	<0.0001	1.84	1.252-2.702	0.0019	1.82	1.195-2.757	0.0052	2.25	1.800-2.815	<0.0001

^a^ Comparisons of categorical variables were analyzed using logistic regression. ^b^ OR was adjusted for all variables in the table. Abbreviations: CI: confidence interval; OR: odds ratio; BMI: Body mass index; CKD: chronic kidney disease; DM: diabetes mellitus.
