# Supplementary material for: The characterization and comorbidities of heterozygous Bardet-Biedl syndrome carriers
Source: Int J Med Sci. 2024 Feb 25;21(5):784–94. doi: 10.7150/ijms.92766 (PMC11008491; doi:10.7150/ijms.92766)
Supplement: Supplementary file 1 — Supplementary tables. [file ijmsv21p0784s1.pdf]

Supplementary Table 1. Stratification by BMI and hyperlipidemia.

Variables	Hyperlipidemia											
	BMI<24 kg/m^2			24≦BMI≦27 kg/m^2			BMI>27 kg/m^2			Overall		
	OR	95% CI	*P* value	OR	95% CI	*P* value	OR	95% CI	*P* value	OR	95% CI	*P* value[a]
Age, years												
Gender												
female	1.00	—	—	1.00	—	—	1.00	—	—	1.00	—	—
male	1.47	1.179-1.823	0.0006	1.56	1.200-2.036	0.0009	1.01	0.763-1.334	0.9491	1.52	1.330-1.745	<0.0001
rs773862084												
CC	1.00	—	—	1.00	—	—	1.00	—	—	1.00	—	—
CA / AA	0.546	0.343-0.868	0.0106	0.93	0.539-1.594	0.7826	0.71	0.431-1.180	0.188	0.67	0.512-0.888	0.0051
rs567573386												
GG	1.00	—	—	1.00	—	—	1.00	—	—	1.00	—	—
GA / AA	0.412	0.119-1.431	0.1629	0.51	0.098-2.633	0.4201	0.26	0.029-2.324	0.2274	0.40	0.165-0.990	0.0476
rs199910690												
CC	1.00	—	—	1.00	—	—	1.00	—	—	1.00	—	—
CT / TT	0.79	0.554-1.134	0.204	0.88	0.585-1.326	0.5425	0.69	0.464-1.011	0.0569	0.86	0.693-1.057	0.1485

[a] Comparisons of categorical variables were analyzed using logistic regression adjusted by age and gender.

Abbreviations: CI: confidence interval; OR: odds ratio; BMI: Body mass index; CKD: chronic kidney disease

Supplementary Table 2. Stratification by BMI and DM.

Variables	DM											
	BMI<24 kg/m^2			24≦BMI≦27 kg/m^2			BMI>27 kg/m^2			Overall		
	OR	95% CI	*P* value	OR	95% CI	*P* value	OR	95% CI	*P* value	OR	95% CI	*P* value[a]
Age, years												
Gender												
female	1.00	—	—	1.00	—	—	1.00	—	—	1.00	—	—
male	2.03	1.603-2.565	<0.0001	1.43	1.092-1.858	0.0091	0.98	0.743-1.299	0.9016	1.79	1.555-2.061	<0.0001
rs773862084												
CC	1.00	—	—	1.00	—	—	1.00	—	—	1.00	—	—
CA / AA	0.59	0.350-0.987	0.0445	0.63	0.355-1.120	0.1155	0.73	0.438-1.205	0.2156	0.64	0.480-0.862	0.0032
rs567573386												
GG	1.00	—	—	1.00	—	—	1.00	—	—	1.00	—	—
GA / AA	0.63	0.182-2.195	0.4702	0.23	0.028-1.937	0.1774	0.28	0.031-2.513	0.2554	0.41	0.155-1.066	0.0674
rs199910690												
CC	1.00	—	—	1.00	—	—	1.00	—	—	1.00	—	—
CT / TT	0.66	0.434-1.002	0.0513	0.82	0.541-1.241	0.346	0.57	0.381-0.847	0.0056	0.72	0.576-0.906	0.0048

[a] Comparisons of categorical variables were analyzed using logistic regression adjusted by age and gender.

Abbreviations: CI: confidence interval; OR: odds ratio; BMI: Body mass index; CKD: chronic kidney disease

Supplementary Table 3. Stratification by BMI and hypertension.

Variables	Hypertension											
	BMI<24 kg/m²			24≦BMI≦27 kg/m²			BMI>27 kg/m²			Overall		
	OR	95% CI	*P* value	OR	95% CI	*P* value	OR	95% CI	*P* value	OR	95% CI	*P* value[a]
Age, years												
Gender												
female	1.00	—	—	1.00	—	—	1.00	—	—	1.00	—	—
male	1.52	1.218-1.905	0.0002	1.32	1.014-1.724	0.0395	1.23	0.931-1.634	0.1443	1.58	1.375-1.812	<0.0001
rs773862084												
CC	1.00	—	—	1.00	—	—	1.00	—	—	1.00	—	—
CA / AA	0.36	0.206-0.624	0.0003	0.83	0.477-1.448	0.5138	0.64	0.382-1.076	0.0922	0.59	0.440-0.787	0.0004
rs567573386												
GG	1.00	—	—	1.00	—	—	1.00	—	—	1.00	—	—
GA / AA	0.49	0.140-1.690	0.257	0.24	0.028-1.981	0.1841	-	-	-	0.28	0.098-0.807	0.0183
rs199910690												
CC	1.00	—	—	1.00	—	—	1.00	—	—	1.00	—	—
CT / TT	0.64	0.432-0.944	0.0244	0.67	0.435-1.022	0.0628	0.76	0.513-1.123	0.1679	0.74	0.592-0.919	0.0067

[a] Comparisons of categorical variables were analyzed using logistic regression adjusted by age and gender.

Abbreviations: CI: confidence interval; OR: odds ratio; BMI: Body mass index; CKD: chronic kidney disease

Supplementary Table 4. Stratification by BMI and hyperlipidemia in females.

Variables	Female											
	BMI<24 kg/m^2			24≦BMI≦27 kg/m^2			BMI>27 kg/m^2			Overall		
	OR[b]	95% CI	*P* value	OR	95% CI	*P* value	OR	95% CI	*P* value	OR	95% CI	*P* value[a]
rs773862084												
CC	1.00	—	—	1.00	—	—	1.00	—	—	1.00	—	—
CA / AA	0.55	0.264-1.162	0.1181	1.54	0.610-3.886	0.3604	0.34	0.113-0.994	0.0487	0.63	0.387-1.021	0.061
Age, years	1.04	1.032-1.054	<0.0001	1.02	0.999-1.035	0.0689	1.02	1.004-1.041	0.0154	1.03	1.025-1.041	<0.0001
DM												
No	1.00	—	—	1.00	—	—	1.00	—	—	1.00	—	—
Yes	4.13	2.889-5.915	<0.0001	4.44	2.727-7.240	<0.0001	6.04	3.616-10.088	<0.0001	5.08	3.991-6.455	<0.0001
CKD												
No	1.00	—	—	1.00	—	—	1.00	—	—	1.00	—	—
Yes	2.43	1.771-3.343	<0.0001	3.92	2.378-6.472	<0.0001	2.12	1.200-3.736	0.0096	2.70	2.130-3.419	<0.0001
rs199910690												
CC	1.00	—	—	1.00	—	—	1.00	—	—	1.00	—	—
CT / TT	1.22	0.746-1.982	0.4338	0.88	0.408-1.910	0.7514	0.78	0.392-1.566	0.4908	1.06	0.755-1.497	0.725
Age, years	1.04	1.031-1.054	<0.0001	1.02	0.999-1.035	0.072	1.02	1.005-1.042	0.0132	1.03	1.024-1.041	<0.0001

DM												
No	1.00	—	—	1.00	—	—	1.00	—	—	1.00	—	—
Yes	4.23	2.957-6.054	<0.0001	4.48	2.750-7.295	<0.0001	5.86	3.525-9.744	<0.0001	5.164	4.061-6.566	<0.0001
CKD												
No	1.00	—	—	1.00	—	—	1.00	—	—	1.00	—	—
Yes	2.51	1.826-3.451	<0.0001	3.86	2.326-6.388	<0.0001	2.29	1.302-4.013	0.004	2.78	2.196-3.529	<0.0001

[a] Comparisons of categorical variables were analyzed using logistic regression.

[b] OR was adjusted for all variables in the table.

Abbreviations: CI: confidence interval; OR: odds ratio; BMI: Body mass index; CKD: chronic kidney disease; DM: diabetes mellitus.

Supplementary Table 5. Stratification by BMI and hyperlipidemia in males.

Variables	Male											
	BMI<24 kg/m^2			24≦BMI≦27 kg/m^2			BMI>27 kg/m^2			Overall		
	OR[b]	95% CI	*P* value	OR	95% CI	*P* value	OR	95% CI	*P* value	OR	95% CI	*P* value[a]
rs773862084												
CC	1.00	—	—	1.00	—	—	1.00	—	—	1.00	—	—
CA / AA	0.84	0.406-1.724	0.6283	1.07	0.491-2.346	0.8597	1.41	0.697-2.844	0.3399	1.07	0.707-1.612	0.7551
Age, years	1.02	1.004-1.030	0.0123	1.01	0.998-1.025	0.0821	1.01	0.999-1.028	0.0633	1.01	1.007-1.022	0.0002
DM												
No	1.00	—	—	1.00	—	—	1.00	—	—	1.00	—	—
Yes	4.00	2.652-6.018	<0.0001	3.34	2.296-4.859	<0.0001	4.71	3.170-7.003	<0.0001	4.34	3.479-5.407	<0.0001
CKD												
No	1.00	—	—	1.00	—	—	1.00	—	—	1.00	—	—
Yes	3.20	2.164-4.743	<0.0001	1.82	1.238-2.663	0.0023	1.84	1.216-2.778	0.0039	2.23	1.787-2.787	<0.0001
rs199910690												
CC	1.00	—	—	1.00	—	—	1.00	—	—	1.00	—	—
CT / TT	0.79	0.381-1.642	0.5293	1.29	0.730-2.267	0.3838	0.97	0.542-1.742	0.9225	1.13	0.807-1.582	0.4767
Age, years	1.02	1.003-1.030	0.0136	1.01	0.999-1.026	0.0787	1.01	1.000-1.029	0.0536	1.01	1.007-1.022	0.0002

DM												
No	1.00	—	—	1.00	—	—	1.00	—	—	1.00	—	—
Yes	4.02	2.667-6.067	<0.0001	3.31	2.279-4.819	<0.0001	4.72	3.172-7.027	<0.0001	4.38	3.509-5.457	<0.0001
CKD												
No	1.00	—	—	1.00	—	—	1.00	—	—	1.00	—	—
Yes	3.16	2.133-4.679	<0.0001	1.83	1.247-2.690	0.002	1.83	1.202-2.780	0.0048	2.25	1.797-2.813	<0.0001

[a] Comparisons of categorical variables were analyzed using logistic regression.

[b] OR was adjusted for all variables in table.

Abbreviations: CI: confidence interval; OR: odds ratio; BMI: Body mass index; CKD: chronic kidney disease; DM: diabetes mellitus.

Supplementary Table 6. Stratification by BMI and DM in females.

Variables	Female											
	BMI<24 kg/m^2			24≦BMI≦27 kg/m^2			BMI>27 kg/m^2			Overall		
	OR[b]	95% CI	*P* value	OR	95% CI	*P* value	OR	95% CI	*P* value	OR	95% CI	*P* value[a]
rs773862084												
CC	1.00	—	—	1.00	—	—	1.00	—	—	1.00	—	—
CA / AA	0.54	0.216-1.356	0.1903	0.50	0.181-1.385	0.1829	1.40	0.528-3.715	0.4986	0.69	0.409-1.155	0.1569
Age, years	1.05	1.037-1.064	<0.0001	1.05	1.033-1.072	<0.0001	1.03	1.01-1.046	0.0023	1.04	1.036-1.053	<0.0001
hyperlipidemia												
No	1.00	—	—	1.00	—	—	1.00	—	—	1.00	—	—
Yes	4.20	2.941-5.984	<0.0001	4.46	2.743-7.261	<0.0001	6.03	3.6.9-10.065	<0.0001	5.51	4.020-6.484	<0.0001
CKD												
No	1.00	—	—	1.00	—	—	1.00	—	—	1.00	—	—
Yes	1.24	0.864-1.780	0.2438	1.09	0.648-1.837	0.7433	1.15	0.656-2.011	0.6276	1.16	0.898-1.486	0.2619
rs199910690												
CC	1.00	—	—	1.00	—	—	1.00	—	—	1.00	—	—
CT / TT	0.63	0339-1.169	0.1429	1.02	0.482-2.155	0.95986	0.70	0.355-1.380	0.3032	0.80	0.552-1.156	0.2337

Age, years	1.05	1.037-1.064	<0.0001	1.05	1.033-1.072	<0.0001	1.03	1.011-1.048	0.0017	1.05	1.036-1.053	<0.0001
hyperlipidemia												
No	1.00	—	—	1.00	—	—	1.00	—	—	1.00	—	—
Yes	4.31	3.023-6.154	<0.0001	4.50	2.766-7.312	<0.0001	5.86	3.527-9.741	<0.0001	5.20	4.094-6.603	<0.0001
CKD												
No	1.00	—	—	1.00	—	—	1.00	—	—	1.00	—	—
Yes	1.23	0.856-1.767	0.2633	1.11	0.653-1.876	0.7055	1.10	0.632-1.919	0.7347	1.15	0.896-1.485	0.2682

[a] Comparisons of categorical variables were analyzed using logistic regression.

[b] OR was adjusted for all variables in the table.

Abbreviations: CI: confidence interval; OR: odds ratio; BMI: Body mass index; CKD: chronic kidney disease; DM: diabetes mellitus.

Supplementary Table 7. Stratification by BMI and DM in males.

Variables	Male											
	BMI<24 kg/m^2			24≦BMI≦27 kg/m^2			BMI>27 kg/m^2			Overall		
	OR[b]	95% CI	*P* value	OR	95% CI	*P* value	OR	95% CI	*P* value	OR	95% CI	*P* value[a]
rs773862084												
CC	1.00	—	—	1.00	—	—	1.00	—	—	1.00	—	—
CA / AA	0.63	0.296-1.343	0.2319	0.65	0.289-1.478	0.3069	0.69	0.335-1.419	0.3129	0.68	0.439-1.039	0.0743
Age, years	1.03	1.017-1.045	<0.0001	1.03	1.018-1.047	<0.0001	1.02	1.005-1.034	0.0095	1.03	1.018-1.033	<0.0001
hyperlipidemia												
No	1.00	—	—	1.00	—	—	1.00	—	—	1.00	—	—
Yes	4.01	2.666-6.030	<0.0001	3.36	2.310-4.887	<0.0001	4.71	3.170-7.003	<0.0001	4.35	3.492-5.423	<0.0001
CKD												
No	1.00	—	—	1.00	—	—	1.00	—	—	1.00	—	—
Yes	1.25	0.826-1.879	0.2944	1.45	0.986-2.136	0.0593	1.85	1.225-2.795	0.0035	1.53	1.219-1.913	0.0002
rs199910690												
CC	1.00	—	—	1.00	—	—	1.00	—	—	1.00	—	—
CT / TT	0.72	0.338-1.514	0.3815	0.81	0.452-1.456	0.4831	0.56	0.305-1.036	0.0649	0.68	0.478-0.97	0.0333
Age, years	1.03	1.018-1.046	<0.0001	1.03	1.018-1.047	<0.0001	1.02	1.004-1.034	0.0104	1.03	1.017-1.033	<0.0001

hyperlipidemia												
No	1.00	—	—	1.00	—	—	1.00	—	—	1.00	—	—
Yes	4.04	2.683-6.075	<0.0001	3.33	2.293-4.845	<0.0001	4.72	3.167-7.015	<0.0001	4.39	3.519-5.469	<0.0001
CKD												
No	1.00	—	—	1.00	—	—	1.00	—	—	1.00	—	—
Yes	1.27	0.840-1.911	0.2591	1.47	0.994-2.159	0.0538	1.73	1.137-2.628	0.0105	1.51	1.202-1.891	0.0004

[a] Comparisons of categorical variables were analyzed using logistic regression.

[b] OR was adjusted for all variables in the table.

Abbreviations: CI: confidence interval; OR: odds ratio; BMI: Body mass index; CKD: chronic kidney disease; DM: diabetes mellitus.
